# Revision process for the *International statistical classification of diseases and related health problems*

**DOI:** 10.2471/BLT.25.294650

**Published:** 2026-07-01

**Authors:** Jonathan ML White, Maaya Kita, Eva Krpelanova, Carine Alsokhn, Maria L Costa, Willem Fibbe, Wolfgang Gaebel, Geoffrey M Reed, Dilani Samarawickrema Lokuhetty, Anthony Woolf, Kameshwar Prasad, Meng Zhang, Rolf-Detlef Treede, Christopher G Chute, Robert Jakob, on behalf of the Medical and Scientific Advisory Committee for the International statistical classification of diseases and related health problems

**Affiliations:** a École de Santé Publique, Université libre de Bruxelles, Route de Lennik 808, Anderlecht, Brussels, 1070, Belgium.; b Division of Data, Analytics and Delivery for Impact, World Health Organization, Geneva, Switzerland.; c Department of Obstetrics and Gynecology, Universidade Estadual de Campinas, São Paulo, Brazil.; d Department of Internal Medicine, Leiden University Medical Center, Leiden, Kingdom of the Netherlands.; e Department of Psychiatry, Heinrich-Heine University, Düsseldorf, Germany.; f Department of Psychiatry, Columbia University Vagelos College of Physicians and Surgeons, New York, United States of America (USA).; g International Agency for Research on Cancer, World Health Organization, Lyon, France.; h Global Alliance for Musculoskeletal Health, Truro, England.; i Department of Neurology, All India Institute of Medical Sciences, New Delhi, India.; j Department of Medical Records, Peking Union Medical College Hospital, Beijing, China.; k Department of Neurophysiology, University of Heidelberg, Germany.; l School of Medicine, Johns Hopkins University, Baltimore, USA.

## Abstract

The *International classification of diseases and related health problems*, *11th revision* (ICD-11) includes an organized system of linked biomedical concepts and hierarchical coding tables called linearizations, of which the most important is the ICD-11 for Mortality and Morbidity Statistics. A publicly accessible web-based platform supports the dynamic maintenance of ICD-11 content, including proposals for modifications, based on scientific evidence and clinical relevance. This paper describes how proposals submitted through the platform are reviewed by the ICD-11 Medical and Scientific Advisory Committee. We analysed World Health Organization data on proposals to modify ICD-11 entities submitted between 2021 and 2025, according to proposal source, subject area, proposal type, time to decision and outcome. The advisory committee reviewed 256 proposals concerning 92% (24/26) of ICD-11 chapters. Proposals were submitted by international bodies, individual practitioners, patient advocacy groups and scholarly societies, with similar acceptance rates across groups. The most common proposal type was the addition of an entry, which had an acceptance rate 65% (81/125); followed by the deletion of an obsolete entity, which had an acceptance rate of 77% (44/57). The mean time to decision was 13.8 months. Overall, 69% (176/256) of proposals were recommended for adoption. These findings indicate that the advisory committee’s review process is functioning adequately, with reasonable acceptance rates following scientific scrutiny. Outreach should be considered to encourage submissions in chapters with few updates between 2021 and 2025. Turnaround time may improve with the expansion of the advisory committee’s membership and clearer guidance on proposal quality.

## Résumé

La *Classification internationale des maladies et des problèmes de santé connexes*, *11^e^ révision* (CIM-11), comprend un système structuré de concepts biomédicaux corrélés et de tableaux de codage hiérarchiques appelés « linéarisations », dont la plus importante est la CIM-11 pour les statistiques de mortalité et de morbidité. Une plateforme web accessible au public permet la mise à jour dynamique du contenu de la CIM-11, y compris les propositions de modification, sur la base de données scientifiques et de la pertinence clinique. Le présent article décrit la manière dont les propositions soumises via la plateforme sont examinées par le Comité consultatif médical et scientifique de la CIM-11. Nous avons analysé les données de l’Organisation mondiale de la Santé relatives aux propositions de modification des entités de la CIM-11 soumises entre 2021 et 2025, en fonction de la source de la proposition, du domaine concerné, du type de proposition, du délai de décision et du résultat. Le comité consultatif a examiné 256 propositions couvrant 92% (24/26) des chapitres de la CIM-11. Celles-ci ont été soumises par des organismes internationaux, des praticiens à titre individuel, des associations de défense des patients et des sociétés savantes, avec des taux d’acceptation similaires entre les différents groupes. Le type de proposition le plus courant était l’ajout d’une entrée, dont le taux d’acceptation s’élevait à 65% (81/125); venait ensuite la suppression d’une entité obsolète, avec un taux d’acceptation de 77% (44/57). Le délai moyen de prise de décision était de 13,8 mois. En tout, 69% (176/256) des propositions ont été recommandées pour adoption. Ces résultats indiquent que le processus d’examen du comité consultatif fonctionne correctement, avec des taux d’acceptation raisonnables à l’issue d’un examen scientifique rigoureux. Il y a lieu d’envisager des actions de sensibilisation afin d’encourager les soumissions dans les chapitres peu mis à jour entre 2021 et 2025. Les délais de traitement pourraient s’améliorer grâce à l’élargissement de la composition du comité consultatif et à des orientations plus claires concernant la qualité des propositions.

## Resumen

La *Clasificación internacional de enfermedades y problemas relacionados con la salud*,* 11.ª revisión* (CIE-11), incluye un sistema organizado de conceptos biomédicos interrelacionados y tablas jerárquicas de codificación denominadas linealizaciones, entre las cuales la más importante es la CIE-11 para Estadísticas de Mortalidad y Morbilidad. Una plataforma web de acceso público permite el mantenimiento dinámico del contenido de la CIE-11, incluidas las propuestas de modificación, basadas en la evidencia científica y la relevancia clínica. En este artículo, se describe el proceso mediante el cual el Comité Asesor Médico y Científico de la CIE-11 revisa las propuestas presentadas a través de la plataforma. Se analizaron los datos de la Organización Mundial de la Salud sobre las propuestas de modificación de entidades de la CIE-11 presentadas entre 2021 y 2025, según su origen, área temática, tipo de propuesta, tiempo hasta la decisión y resultado. El comité asesor examinó 256 propuestas correspondientes al 92% (24/26) de los capítulos de la CIE-11. Las propuestas fueron presentadas por organismos internacionales, profesionales individuales, organizaciones de defensa de los pacientes y sociedades científicas, con tasas de aceptación similares entre los distintos grupos. El tipo de propuesta más frecuente fue la incorporación de una entrada, con una tasa de aceptación del 65% (81/125), seguida de la eliminación de una entidad obsoleta, con una tasa de aceptación del 77% (44/57). El tiempo medio hasta la adopción de una decisión fue de 13,8 meses. En conjunto, se recomendó la adopción del 69% (176/256) de las propuestas. Estos resultados indican que el proceso de revisión del comité asesor funciona adecuadamente, con tasas de aceptación razonables tras la correspondiente evaluación científica. Deberían promoverse acciones de difusión para fomentar la presentación de propuestas en los capítulos que recibieron pocas actualizaciones entre 2021 y 2025. El tiempo de tramitación podría reducirse mediante la ampliación de la composición del comité asesor y la elaboración de orientaciones más claras sobre la calidad de las propuestas.

## ملخص

يتضمن *التصنيف الدولي للأمراض والمشاكل الصحية ذات الصلة، الطبعة الحادية عشرة (ICD-11) *، نظامًا منسقًا من المفاهيم الطبية الحيوية المترابطة، وجداول الترميز الهرمية المعروفة باسم "التسلسلات الخطية"، وأهمها إحصائيات الوفيات والإصابة بالأمراض وفقًا لتصنيف ICD-11. هناك منصة على الويب متاحة للجمهور، تدعم الصيانة الديناميكية لمحتوى ICD-11، بما في ذلك مقترحات التعديلات، استنادًا إلى الأدلة العلمية والأهمية الإكلينيكية. تصف هذه الورقة البحثية كيفية مراجعة المقترحات المقدمة عبر المنصة بواسطة اللجنة الاستشارية الطبية والعلمية لتصنيف ICD-11. قمنا بتحليل بيانات منظمة الصحة العالمية حول مقترحات تعديل كيانات ICD-11 المقدمة بين عامي 2021 و2025، وفقًا لمصدر المقترح، ومجاله، ونوعه، والمدة الزمنية اللازمة لاتخاذ القرار، والنتيجة. راجعت اللجنة الاستشارية 256 مقترحًا تتعلق بنسبة %92 (24/26) من فصول ICD-11. تم تقديم المقترحات بواسطة هيئات دولية، وممارسين أفراد، وجماعات مناصرة للمرضى، وجمعيات علمية، وكانت معدلات قبولها متقاربة بين مختلف المجموعات. وكان النوع الأكثر شيوعًا من المقترحات هو إضافة مدخل جديد، حيث بلغ معدل قبوله %65 (81/125)؛ يليه حذف كيان قديم، بمعدل قبول بلغ %77 (44/57). وبلغ متوسط مدة اتخاذ القرار 13.8 شهرًا. وبشكل عام، تمت التوصية بنسبة %69 من المقترحات (176/256) للاعتماد. تشير هذه النتائج إلى أن عملية مراجعة اللجنة الاستشارية تعمل بكفاءة، مع معدلات قبول معقولة بعد التدقيق العلمي. يتم النظر في التواصل بهدف تشجيع تقديم المقترحات في الفصول التي لم تشهد تحديثات كثيرة بين عامي 2021 و2025. وقد تتحسن مدة الاستجابة مع توسيع عضوية اللجنة الاستشارية وتوفير إرشادات أوضح بشأن جودة المقترحات.

## 摘要

《国际疾病及相关健康问题统计分类（第 11 版）》(ICD-11) 包含一套完善的关联生物医学概念体系与分层编码表，这类编码表称为线性分类表；其中最重要的是 ICD-11 死亡与发病统计专用线性分类表。一款可公开访问的线上平台支持对 ICD-11 内容进行动态维护，使用者可根据科学依据与临床相关性提出修订提案。本文介绍由 ICD-11 医学科学咨询委员会对通过该平台提交的提案进行审核的流程。我们分析了世卫组织 2021 至 2025 年间收到的 ICD-11 类目修订提案相关数据，分析维度包括提案提交方、学科领域、提案类型、决议周期及处理结果。咨询委员会共审核 256 份提案，覆盖 ICD-11 中 92% (24/26) 章节内容。提案提交主体包括国际机构、个体临床从业人员、患者权益组织及学术协会，各类主体提交提案的采纳率相近。最常见的提案类型为新增类目，该类提案采纳率为 65% (81/125)；其次是删除过时类目，此类提案采纳率达 77% (44/57)。平均审核决议周期为 13.8 个月。总体而言，69% (176/256) 提案获得采纳推荐。上述研究结果表明，咨询委员会的审核流程运行良好，经科学审查后的提案采纳率处于合理区间。针对 2021 至 2025 年间修订提案较少的 ICD-11 章节，应开展推广动员工作以鼓励相关机构提交修订提案。通过扩充咨询委员会成员规模、出台更清晰的提案质量规范，或可缩短提案处理周期。

## Резюме

*Международная классификация болезней и проблем, связанных со здоровьем*, *11-го пересмотра* (МКБ-11) включает в себя организованную систему связанных биомедицинских концепций и иерархических таблиц кодирования, называемых линеаризациями. Важнейшей из них является МКБ-11 для ведения статистики смертности и заболеваемости. Общедоступная веб-платформа поддерживает динамическое обновление содержимого МКБ-11, включая предложения по изменениям на основе научных данных и клинической значимости. В данном документе описывается, как Медицинский и научно-консультативный комитет по МКБ-11 рассматривает предложения, поступающие через эту платформу. Авторы проанализировали данные Всемирной организации здравоохранения по предложениям об изменении записей МКБ-11, поданных в период с 2021 по 2025 год. Анализ проводился в соответствии с источником предложения, предметной областью, типом предложения, временем принятия решения и результатом. Консультативный комитет рассмотрел 256 предложений, касающихся 92% (24 из 26) разделов МКБ-11. Предложения поступали от международных организаций, отдельных практикующих врачей, групп по защите прав пациентов и научных обществ, при этом доля одобренных заявок была сходной для всех групп. Чаще всего предлагалось добавить новую запись, при этом показатель одобрения составил 65% (81 из 125). Далее следовало удаление устаревшей записи с показателем одобрения 77% (44 из 57). Среднее время принятия решения составило 13,8 месяца. В целом к принятию было рекомендовано 69% (176 из 256) предложений. Эти результаты указывают на то, что процесс рассмотрения консультативным комитетом функционирует надлежащим образом и демонстрирует приемлемые показатели одобрения после научной проверки. Следует рассмотреть возможность проведения информационно-разъяснительной работы для стимулирования подачи предложений по разделам, которые редко обновлялись в период с 2021 по 2025 год. Время обработки заявок может сократиться за счет расширения состава консультативного комитета и предоставления более четких рекомендаций по качеству предложений.

## Introduction

The *International statistical classification of diseases and related health problems* (ICD) provides a standardized and comprehensive framework for recording clinical information, analysing and reporting health data and statistics, and supporting decision-making and intervention algorithms. In doing so, ICD enhances the accuracy of reporting disease diagnoses and treatments. The latest version of the ICD, the 11th revision (ICD-11), was adopted by the Seventy-second World Health Assembly in May 2019 and came into effect in January 2022.^1^^,^^2^ Unlike its predecessors, ICD-11 comprises a new semantic network of biomedical concepts (known as the foundation), a clinical terminology, a variety of tables of hierarchical codes and a formal ontology.^3^

Concepts covered by the ICD-11 foundation, as well as the hierarchical relationships among them, may change with newly published scientific data and evolving clinical practice, resulting in significant changes becoming necessary in the hierarchy and placement of entities. Accordingly, an ICD-11 maintenance platform was created to keep ICD-11 up to date for the benefit of those using it. The maintenance platform ensures that the foundation can be adapted rapidly (Box 1) according to scientific and clinical advances and evolving public health needs, such as monitoring epidemics and enabling appropriate public health responses, while keeping ICD-11 linearizations as stable as possible. This global process for integrating scientific advances into the ICD is essential for global public health for both communicable and noncommunicable diseases and health conditions. Linearization refers to an extraction of a particular hierarchical coding table from the foundation. For the World Health Organization’s (WHO) purposes, the most important linearization is the ICD-11 for Mortality and Morbidity Statistics, which is used by Member States as a basis for reporting of health statistics to WHO.

Box 1 **Medical and Scientific Advisory Committee of the WHO Family of International Classifications Network**

The Medical and Scientific Advisory Committee was established in 2016 as part of the WHO Family of International Classifications Network, which oversees all aspects of maintenance and implementation of ICD-11 and related classifications.^4^ The advisory committee performs the scientific evaluation of proposals received in the public ICD-11 maintenance platform (hereafter, proposals) for changes to the foundation of the ICD-11. To ensure the scientific validity and clinical relevance of inputs received during the ICD maintenance process, the advisory committee provides clinical and scientific advice on proposed changes from global users and may itself also recommend changes. The process is similar to peer review for scientific publications but also considers additional aspects for classification purposes, such as global stakeholder consensus and statistical continuity with previous versions.

Proposals are continuously received from individuals, patient advocacy groups, national and international health agencies and scholarly societies. For approved proposals, the advisory committee adapt the foundation and the Mortality and Morbidity Statistics in accordance with scientific and practice advances, while at the same time keeping the statistical rendering stable.

ICD-11: *International statistical classification of diseases and related health problems*, 11th revision; WHO: World Health Organization.

WHO updates the Mortality and Morbidity Statistics in response to accepted proposals each year. Proposals can be submitted to WHO’s web-based family of international classifications maintenance platform by any person or entity; the proposals are reviewed by the Medical and Scientific Advisory Committee (hereafter the advisory committee; Box 1). The collaborative refinement of ICD-11 is an important benefit of its inherently flexible nature.^5^

In this article, we describe our internal procedure to ensure transparency for those wishing to submit a proposal. We also identify opportunities to optimize the process for reviewers and proposers by auditing our data to identify potential bias in outcomes associated with proposer identity and by evaluating the timeliness of process, all with quality control in mind. 

## ICD-11 proposal flow 

Proposals submitted to the advisory committee are categorized as an addition of entity, a deletion of entity, a content enhancement, a complex hierarchical change or a postcoordination. Adding or moving an entity affects its higher-level (parent) and lower-level (child) entities within the hierarchy. A deletion does not eliminate an entity entirely from the foundation, instead, the entity is marked as deprecated, ensuring that it remains searchable as a historical synonym. A content enhancement changes aspects of an entity such as its title, brief description or synonyms; it does not remove the entity, add a new entity or change its place in the hierarchy. A complex hierarchical change involves changing an entity’s location in the hierarchy and/or its relationships with parent or child entities. Postcoordination proposals are not reviewed by the advisory committee.

**Fig. 1:**
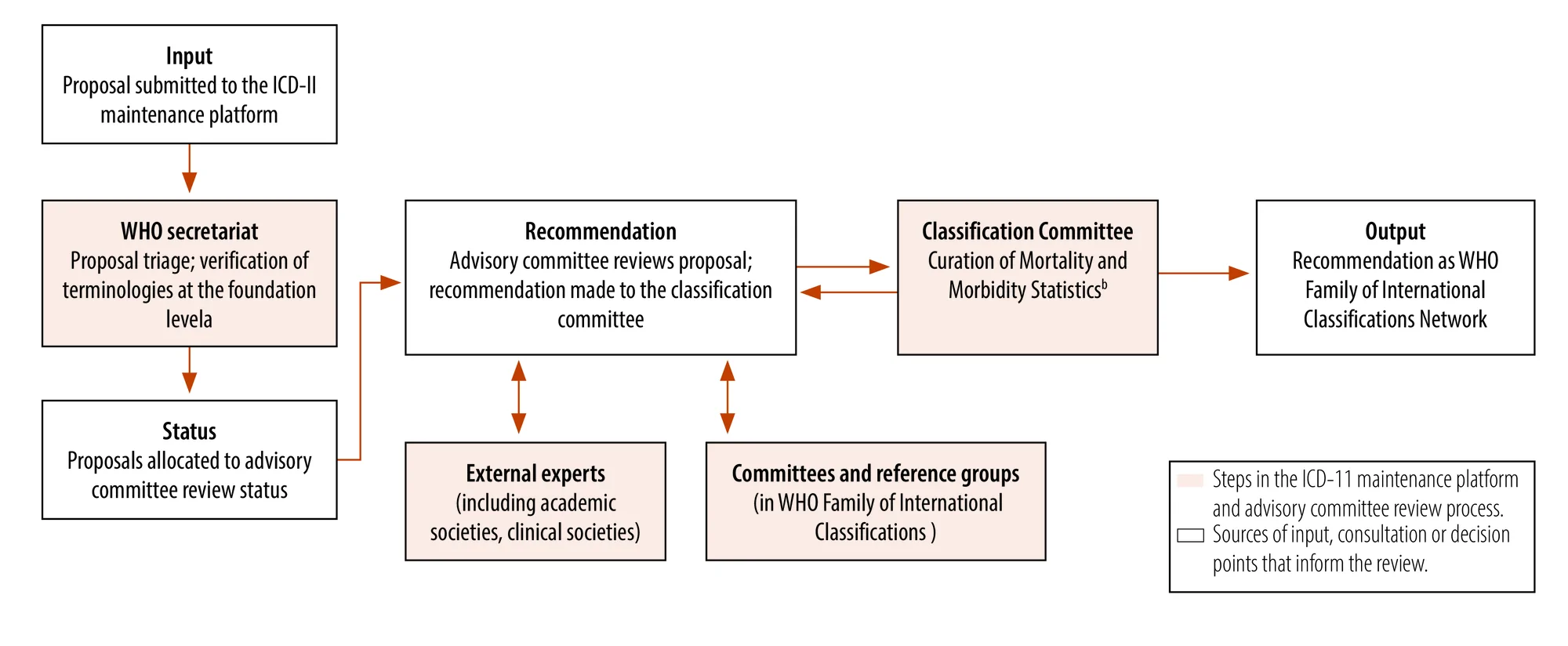
Input to output flow for proposed adaptations to the *International statistical classification of diseases and related health problems* reviewed by the Medical and Scientific Advisory Committee

Upon submission, proposals undergo an initial triage by members of the WHO secretariat to assess completeness, for example, inclusion of supporting references (Fig. 1). The conceptual boundaries and guiding principles for changes to the ICD foundation are based on published scientific references and documentation reflecting international professional consensus. The criteria are applied across all potential proposal categories. Some minor proposals, such as corrections of typographical errors or the addition or removal of synonyms or exclusions, do not require advisory committee review. Proposals that do not meet the minimum submission requirements are returned to the proposer for clarification. If a proposal is deemed complete and requires review, it is referred to the advisory committee. This committee’s role is to determine whether a proposal is scientifically and clinically accurate as well as relevant to the foundation. Where necessary, advisory committee members may consult external experts, other groups within the WHO Family of International Classifications Network (hereafter, the classifications network) and WHO technical departments.

Advisory committee: Medical and Scientific Advisory Committee; classifications committee: Classification and Statistics Advisory Committee; ICD-11: *International statistical classification of diseases and related health problems*, 11th revision; WHO: World Health Organization.

^a^ The ICD-11 foundation is the underlying knowledge base of ICD-11 content. Proposed changes are reviewed for consistency with clinical meaning and use, current biomedical knowledge and the formal relationships and classification logic that structure the ICD-11 foundation.

^b^ Curation of the Mortality and Morbidity Statistics refers to reviewing proposals and linearization from a classification and statistical perspective.

Notes: The advisory committee is part of the WHO Family of International Classifications Network. It comprises collaborating centres and other working groups and makes recommendations to Classification and Statistics Advisory Committee for consideration of adoption into ICD-11 Mortality and Morbidity Statistics linearization. The classification committee is responsible for implementing coding rules and classification principles.

Proposals forwarded to the advisory committee involve changes to the foundation that may or may not subsequently be visible in a linearization such as the Mortality and Morbidity Statistics. In some cases, detailed content deep within the foundation hierarchy is not exposed in the linearization and therefore does not display as a codable entity.^3^ Such content, however, remains searchable and can thereby enhance the precision and clinical applicability of the corresponding parent codable rubric. Whether a concept should be made visible in a linearization is determined by the Classification and Statistics Advisory Committee (hereafter, the classifications committee), the body responsible for implementing coding rules and classification principles. By distinguishing technical content from what is included in the codable system for the Mortality and Morbidity Statistics, ICD-11 attains the flexibility and depth characteristic of a terminology.

Examples of proposals that resulted in changes to Mortality and Morbidity Statistics linearization in the ICD-11 since its approval in 2019 include reclassification of infectious agents (proposal #2H6S); new concepts in pathophysiology (#2A2Q); new developments in diagnosis (#2H2G) and therapy (#2Q9X); and new genetic insights into disease classification (#2W3F), all of which were supported with published scientific evidence.^6^^–^^10^ All proposals can be viewed via the WHO classifications network maintenance platform.^11^ Further to the Mortality and Morbidity Statistics linearization, additional linearizations can be extracted from the foundation for specific needs, such as the ICD-11 classification of dermatological diseases^12^ and the clinical descriptions and diagnostic requirements for ICD-11 mental, behavioural and neurodevelopmental disorders.^13^

### Scientific reviewing and consensus process of the advisory committee

The advisory committee, appointed by WHO, comprises 22 members representing various geographical regions, clinical settings and classification committee co-chairs. The advisory committee members serve as experts and are also part of the classifications network; external experts and relevant regional offices or technical teams at WHO headquarters are contacted if necessary. The advisory committee has two co-chairs and is supported by the secretariat. Meetings take place monthly online, with an annual plenary conference during the classifications network annual meeting. The members’ declarations of conflict of interest are updated annually following WHO standard procedures, an important feature to ensure transparency.

**Fig. 2:**
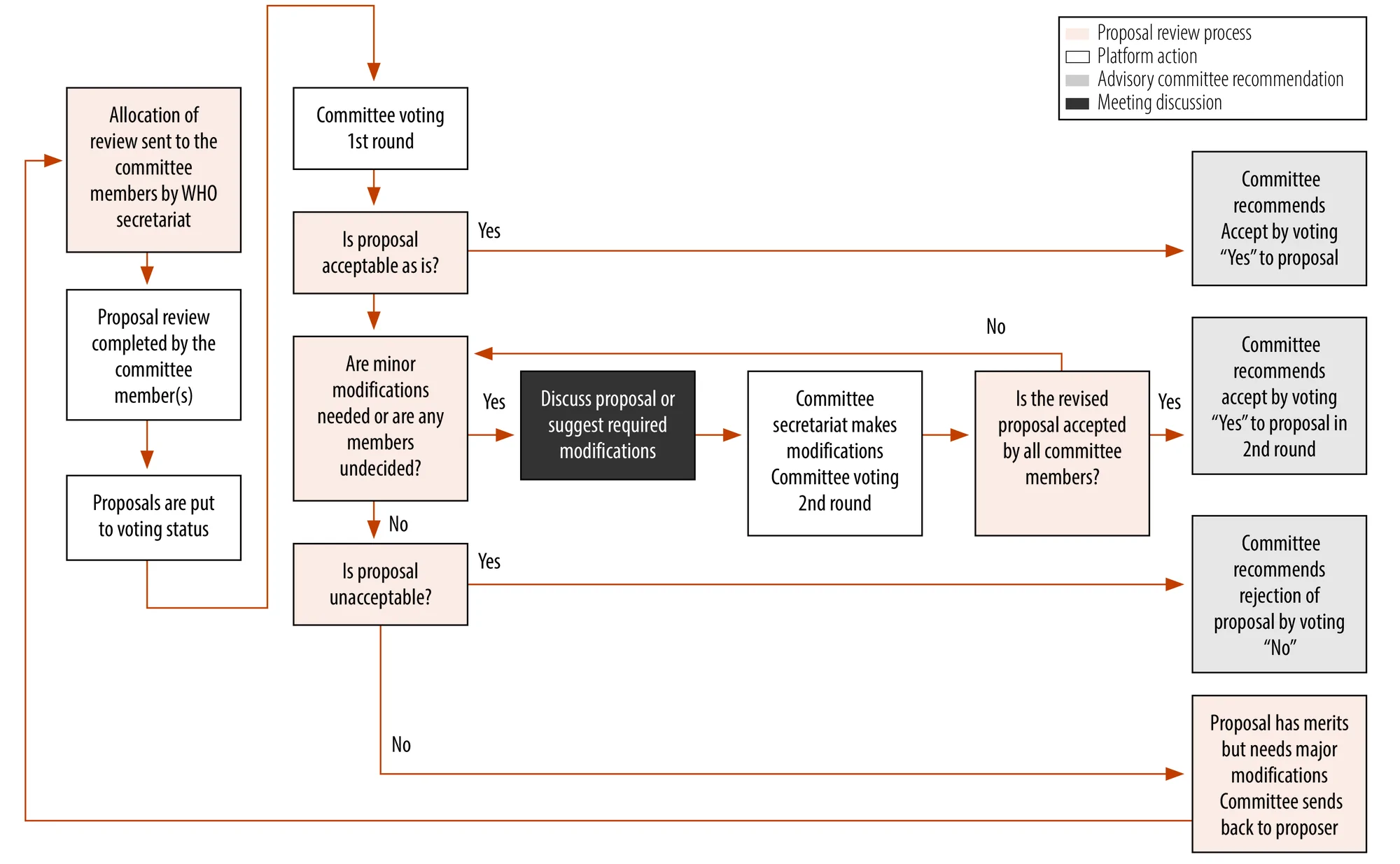
Workflow of the Medical and Scientific Advisory Committee for proposed adaptations to the *International statistical classification of diseases and related health problems*

The advisory committee’s workflow is detailed in Fig. 2. Once a proposal is referred to this committee, an appropriate member is assigned to review it and provide a recommendation to accept it (with or without modifications), reject it or send it back to the proposer with specific comments. The review also involves identifying the proposal’s source and any potential association with specific secondary interests.

WHO: World Health Organization.

Note: The committee refers to the Medical and Scientific Advisory Committee.

Once the review is complete, it is shared with the advisory committee members for voting. If further discussion is needed, the reviewer presents the proposal at a meeting. A second vote is then held and the outcome is determined. Recommendations are typically made by consensus among all members. A summary paragraph describing the advisory committee’s recommendation following voting is then published on the public section of the maintenance platform.

If the advisory committee recommends accepting a proposal, it is forwarded to the classification committee, which evaluates it from the perspective of classification and health statistics. The classification committee determines what implications, if any, the proposal has for the Mortality and Morbidity Statistics and shares its assessment and recommendation with responsible officers at WHO. An example of a simple proposal that was accepted is the change in nomenclature of the entity previously known as monkeypox to mpox in 2023.^14^

## Proposal characteristics

To improve the understanding of the proposals reviewed by the advisory committee and their outcomes, we obtained data on all proposals submitted to the committee for which the review process was completed between January 2021 and December 2025 inclusive. We extracted the proposals within this time frame from the ICD-11 maintenance platform in May 2026, including the source and type of proposal, the time taken for review and the final outcome. We also included documentation from internal advisory committee meetings relating to those proposals. 

To assess if there were any statistical differences in acceptance rates, we used Fisher’s exact test for all comparisons due to small cell counts in several categories, with the Freeman-Halton extension to accommodate larger contingency tables. The Holm correction was applied for pairwise comparisons when necessary to control the family-wise error rate. The overall acceptance rate was calculated as the number of proposals accepted with or without modification divided by the total number reviewed, multiplied by 100.

Between January 2021 and December 2025, 256 proposals underwent complete reviews by the advisory committee. The proposals came from a range of sources: 42% (108/256) were submitted from national or international bodies, such as classification network members and health ministries; 36% (92/256) from individuals, typically researchers or practitioners; 14% (36/256) from patient advocacy groups and 8% (20/256) from scientific or professional societies.

Over the five-year period, proposals most often related to chapters 1 (certain infectious or parasitic diseases), 2 (neoplasms), 8 (diseases of the nervous system) and 24 (factors influencing health status). Two chapters received no proposals at all: 25 (codes for special purposes) and 26 (traditional medicines).

The number of proposals on which the advisory committee reached agreement each year remained fairly stable over time. The acceptance rates for the four categories of proposals referred between 2021 and 2025 are presented in Table 1. The advisory committee accepted 69% (176/256) of all proposals, with annual category-specific acceptance rates ranging from 25% (1/4) to 88% (14/16). Between 13 and 56 proposals were accepted each year, and the average time taken to reach agreement was 13.8 months with a standard deviation of 8.0 months. Of the types of proposals the committee received, additions were the most frequent and the committee accepted 65% (81/125). Deletions were the next most frequent, with an acceptance rate of 77% (44/57) and were primarily concerned with entities considered obsolete. Complex hierarchical changes and content enhancements were less frequent, with acceptance rates of 73% (29/40) and 65% (22/34), respectively. 

**Table 1:** Acceptance rate of different types of proposals for adaptations to the *International statistical classification of diseases and related health problems*, 2021–2025

Type of proposal	2021	2022	2023	2024	2025	2021–2025
**Addition of entity**						
No. reviewed	16	14	29	35	31	125
No. accepted (%)	9 (56)	7 (50)	17 (59)	23 (66)	25 (81)	81 (65)
**Deletion of entity**						
No. reviewed	24	4	10	3	16	57
No. accepted (%)	19 (79)	1 (25)	8 (80)	2 (67)	14 (88)	44 (77)
**Content enhancement**						
No. reviewed	4	4	11	9	6	34
No. accepted (%)	1 (25)	2 (50)	8 (73)	7 (78)	4 (67)	22 (65)
**Complex hierarchical change**						
No. reviewed	3	4	10	8	15	40
No. accepted (%)	1 (33)	3 (75)	7 (70)	5 (63)	13 (87)	29 (73)

Acceptance rates did not differ significantly across years for any proposal type: additions (*P*-value: 0.198), deletions (*P*-value: 0.116), content enhancement (*P*-value: 0.448) and complex hierarchical change (*P*-value: 0.325). However, the small cell counts in several categories may have limited the ability to detect true differences.

Table 2 summarizes the outcomes of types of proposals reviewed by the advisory committee from 2021 to 2025 and shows the trends in agreement over this period. The percentage of proposals accepted outright was 39% (101/256) from 2021 to 2025, whereas proposals accepted with modification was 29% (75/256). Proposals returned to the proposer for further information or clarification accounted for a minority of 9% (22/256) over the same period; these proposals may ultimately be accepted after modification. In contrast, rejections are final, although there is no mechanism to prevent new proposals on the same entity. Rejections constituted 23% (58/256) of the advisory committee’s decisions.

**Table 2:** Outcomes of proposals of all types for adaptations to the *International statistical classification of diseases and related health problems*, 2021–2025

Outcome of the proposal after advisory committee discussion	No. (%)					
	2021 (*n* = 47)	2022 (*n* = 26)	2023 (*n* = 60)	2024 (*n* = 55)	2025 (*n* = 68)	2021–25 (*n* = 256)
No. accepted (%)	14 (30)	8 (31)	24 (40)	22 (40)	33 (49)	101 (39)
No. accepted with modification (%)	16 (34)	5 (19)	16 (27)	15 (27)	23 (34)	75 (29)
Total accepted with or without modification (%)	30 (64)	13 (50)	40 (67)	37 (67)	56 (82)	176 (69)
No. rejected (%)	14 (30)	7 (27)	16 (27)	13 (24)	8 (12)	58 (23)
No. returned to proposer (%)^a^	3 (6)	6 (23)	4 (7)	5 (9)	4 (6)	22 (9)

^a^ Proposals that do not meet the minimum submission requirements are returned to the proposer for clarification.

Note: inconsistencies arise in some values due to rounding.

Overall acceptance rates, with or without modification, across the four types of proposer were also consistent, ranging between 67% and 69%. 

### Examples of ICD-11 curation

To illustrate how curation of the ICD-11 foundation and the Mortality and Morbidity Statistics works in practice, Box 2 provides an example in which a code was changed to synonym of an existing code and, on the advice of the advisory committee, a further code was deprecated. The creation of the proposal started with the special interest group on complex regional pain syndrome of the International Association for the Study of Pain, which held a consensus workshop during an international congress in 2019 to resolve questions and ambiguities in the diagnostic criteria for this syndrome. The group published its research consensus in a peer-reviewed journal^7^ and formulated it into a proposal, submitted to the ICD-11 proposal platform (proposals #2A2Q and #2C5V).^11^ The proposals were supported by the International Association for the Study of Pain’s task force for the classification of chronic pain and its neuropathic pain special interest group, the American Autonomic Society and the International Society of Physical and Rehabilitation Medicine. The proposal met the criteria for quality, rationale and background, as provided in the published paper,^7^ in the supporting evidence^15^^–^^19^ and as reflected in the interdisciplinary international consensus.^7^ Because the screening by the WHO secretariat identified that major scientific medical issues from the proposal would, if accepted, alter the foundation considerably, the proposal required formal review by the advisory committee. After internal review, the advisory committee discussed the proposal #2A2Q in its session on 19 October 2021 and modified it by deprecating the two historical terms, causalgia and algoneurodystrophy, to searchable synonyms for two subtypes of the syndrome rather than codable entities. One of the synonyms was the subject of a separate proposal, allocated to the classification committee; because its co-chairs are ex officio members of the advisory committee, the two work streams were easily joined for this specific case, though it is only rarely necessary. The 2022 annual update of ICD-11 included the proposed and modified changes.

Box 2 **Example of a complex hierarchical change reflecting innovation in medicine based on evidence and consensus: complex regional pain syndrome**

***Proposal on complex regional pain syndrome - #2A2Q***

The advisory committee discussed proposal #2A2Q on complex regional pain syndrome to move the parent focal or segmental autonomic disorder (8D8A) and primary parent to chronic primary pain (MG30.0). The advisory committee agreed to accept the proposal, with minor modifications and clarifications to the content of the proposal:

- The higher-level (parent) code – complex regional pain syndrome – will be moved from the nervous system chapter to the signs and symptoms chapter; and the two lower-level (child) codes under it – complex regional pain syndrome type 1 and complex regional pain syndrome type 2 – will become noncodable entities in the foundation. When moved to the new parent code, these entities would have seven-character codes within the hierarchy, which is not allowed. The advisory committee agreed that these entities are no longer clinically relevant but will be kept in the foundation to preserve the legacy of the previous version of the ICD.
- The modification to the proposal is to change the code causalgia to a synonym of complex regional pain syndrome type 2 rather than a child of it.
- In addition, the advisory committee suggested listing the code algoneurodystrophy as a deprecated synonym of complex regional pain syndrome type 1 (described in proposal #2C5V, assessed by the classification committee).

This update reflects a long evolution of the understanding of chronic pain conditions following physical trauma. Description of causalgia date back to Weir Mitchell’s observations in soldiers with gunshot wounds in the American Civil War,^15^ while reflex dystrophy (algoneurodystrophy) was described by Paul Sudeck in surgical patients at the Eppendorf University Hospital in Hamburg.^16^ For about a century, the autonomic nervous system was considered to play a key role in the pathophysiology of these two conditions since René Leriche discovered the beneficial therapeutic effects of sympathectomy.^17^ After decades of debate about whether sympathetic hyperactivity or adrenoreceptor supersensitivity is more important, the concept of complex regional pain syndrome evolved as a syndrome with four dimensions,^18^ where the autonomic nervous system is involved in some of the clinical presentations, but sensory signs and symptoms are related more to neurogenic inflammation and central sensitization. These characteristics make complex regional pain syndrome a candidate for classification as chronic primary pain.^19^

ICD: *International statistical classification of diseases and related health problems*Note: Adapted from the minutes of the advisory committee meeting on 19 October 2021: Summary of advisory committee discussion.

A further example, regarding uterus donation (proposal #2Q9X),^11^ concerned the addition of a child entity to the parent entity for donors of organs or tissues. The advisory committee reviewer recommended acceptance, noting that uterine transplantation is a well-established intervention. However, the proposal also prompted a broader review of the organ transplantation area of the foundation, reflecting that transplantation now encompasses not only organs but also tissues and cells. The advisory committee therefore expanded the entities into three groups: conventional organs, tissues and cells. Broadening the categories provides scope for additional items to be added in future; this example illustrates that the curation process is not simply one of approving or rejecting proposals. The process is interactive and proposals may stimulate change to the foundation to allow it to match the best medical understanding more closely, and to build in flexibility for the future to include new entities in a logical fashion.

## Discussion

Here we observed that most proposals submitted to the platform were received from government entities involved in public health or coding, both within and external to the WHO classification network. The second largest contribution was from individuals; there was no evidence of differential rates of acceptance between individuals or patient advocacy groups compared with scholarly societies or international bodies. Nor was there evidence of widespread market-driven pressures on ICD-11 disease classification; this finding may be because, at the time of analysis, agencies such as the United States Food and Drug Administration or the European Medicines Agency were still using the previous ICD-10. Chapters for which no proposals have been submitted may reflect a stable field of medicine or indifference of its actors. The advisory committee will consult stakeholders to probe the reasons for this.

The largest number of proposals involved either addition or deletion of an entity, mostly not affecting the Mortality and Morbidity Statistics. Thus, division of tasks between advisory committee (enrichment of foundation) and the classification committee (coding continuity in the Mortality and Morbidity Statistics) appears, in principle, to be effective. Acceptance with or without simple modifications following review by the advisory committee was high, approximately two thirds, which may encourage the submission of proposals in future.

The review of 256 proposals by the advisory committee during a five-year period illustrates its capacity to modify foundation content in step with changes in biomedical science. 

On average, the advisory committee takes just over a year to reach a decision, highlighting the complexity of the issues under consideration and the need for thorough review before voting. Committee members serve on a voluntary basis and there is a limit as to how many reviews each member can perform. Furthermore, while it is uncertain how much the coronavirus disease 2019 pandemic affected submissions of proposals, the pandemic certainly affected availability of some of the committee members due to a diverted public health focus. In light of these findings, WHO is expanding the number of advisory committee members to address timeliness, and in anticipation of increased numbers of proposals as ICD-11 is adopted by more countries and becomes more visible internationally.

The advisory committee is a key resource for users of ICD-11 to ensure the quality of the foundation, which helps address health priorities. In addition to reviewing proposals, the advisory committee works closely with other committees and reference groups within the classifications network. By making recommendations that reflect the latest medical and scientific advances, the advisory committee helps ensure that ICD-11 remains current and clinically relevant. The data presented suggest that while there is minor variability in acceptance rates year-by-year, all of the proposal types have relatively high acceptance rates over time, suggesting progressive refinement of the structure and content of the ICD-11.

The advisory committee is mindful of the sometimes counterproductive or unnecessarily costly consequences of expanding diagnostic categories.^20^ The careful review process ensures academic rigour by relying on declaration of published evidence for the proposal and affiliation of the proposer, as well as demonstration of consensus in the field and the broad panel of experts from different medical specialties, linked with WHO’s impartiality. The fact that over a fifth of proposals were rejected demonstrates the committee’s focus on quality and clinical relevance, as well as a sign that the advisory committee is not afraid to make rejections, no matter who submits them. Grounds for rejection include reasons such as bias in the interpretation of the published data or a lack of international consensus about a controversial topic.

## Conclusions

The advisory committee’s work in curating ICD-11 is essential to keep the classification up to date and clinically relevant. According to our data, the review process is fit for purpose. However, the submission process could be made even more user-friendly by collecting feedback from proposers. Moreover, providing a clear outline of the components of an ideal proposal, which should cover scientific rationale, stakeholder engagement and clinical impact, would likely improve submission quality and further streamline the committee’s review workflow. Finally, information provided in this article might help proposers in their preparation and could increase the chances of obtaining a positive review of their proposal. To optimize ICD-11 curation, future research should capture the complete scope of ICD-11 curation activities. Furthermore, there is a need for a more robust declaration of the proposer’s potential conflicts of interest and their exact affiliations. 

The advisory committee’s contributions within WHO’s classification network remain a key part of WHO’s standard-setting role. By integrating medical and scientific knowledge with research, the advisory committee helps ensure that ICD-11 is grounded in evidence-based policy-making.
